# Traitement chirurgical des fractures articulaires du calcanéum par plaque vissée

**DOI:** 10.11604/pamj.2015.20.291.4635

**Published:** 2015-03-25

**Authors:** Nassreddine Hammou, Hatim Abid, Mohammed Shimi, Abdelhalim El Ibrahimi, Abdelmajid El Mrini

**Affiliations:** 1 Service de Chirurgie Ostéo-Articulaire B4, CHU Hassan II, Fès, Maroc

**Keywords:** Calcanéum, fracture thalamique, ostéosynthèse, plaque vissée, Calcaneum, thalamic fracture, osteosynthesis, bone plate

## Abstract

Les fractures du calcanéum sont peu fréquentes mais le plus souvent graves. Le traitement chirurgical par plaque vissée est ardemment défendu. L'objectif de notre travail rétrospectif est d’évaluer les résultats du traitement chirurgical des fractures articulaires du calcanéum à travers une série de 12 patients opérée aux service d'orthopédie du CHU Hassan II de Fès sur une durée de 3 ans, et les comparer aux données de la littérature. L’âge moyen dans notre série était de 34 ans, le geste opératoire était réalisé au 7^ème^ jour. Tous nos patient ont bénéficie d'une réduction à foyer ouvert avec une ostéosynthèse par plaques vissées. Le recul moyen était de 12 mois et les résultats fonctionnels ont été évaluer selon le score de Kitaoka.

## Introduction

Les fractures du calcanéum sont peu fréquentes, elles représentent entre 1 et 2% de toutes les fractures. Dans 75% des cas, ces fractures sont articulaires. Elles touchent l'articulation sous talienne et exposent à un risque important d'arthrose. Ce sont des fractures à évolution longue avec un nombre non négligeable de complications. Elles relèvent souvent d'un traitement chirurgical qui vise à restaurer l'anatomie de l'articulation sous-talienne. Les auteurs rapportent dans ce travail, les résultats d'une série de 12 cas de fractures articulaires du calcanéum, traitées par un abord externe, une réduction, et une ostéosynthèse par une plaque anatomique, avec révision clinique et radiologique jusqu'au dernier recul.

## Méthodes

C'est une étude rétrospective étalée sur 3 ans (entre janvier 2009 et décembre 2011) d'une série de 12 patients, opérés au service de traumatologie et orthopédie B4 du CHU Hassan II de Fès, pour des fractures articulaires du calcanéum traitées par une plaque anatomique, avec un recul moyen de 18 mois. Les fractures étaient analysées, après des radiographies standard (incidences retro-tibial et cheville de face et de profil) et un scanner de l'arrière pied, selon la classification de Duparc et celle d'Uthésa. Nos résultats ont été évalués grâce à l'angle de Bohler calculé en pré et post opératoire et le score fonctionnel de Kitaoka.

## Résultats

L’âge moyen de notre série était de 34 ans (18-44 ans), avec une prédominance masculine. 75% des patients étaient victimes d'un accident de travail. Toutes les fractures étaient fermées. Une lésion osseuse associée a été notée dans 12% des cas à type de fractures du pilon tibial homolatéral et du rachis lombaire. Selon la classification de Duparc, les fractures étaient de type III dans 42% des cas et de type IV dans 58% des cas. L'angle de Bohler était négatif dans la moitié des cas et nul chez 5 de nos patients. Au scanner, L'enfoncement thalamique était de type vertical dans 42% des cas, horizontal dans 8% des cas et mixte dans 50% des cas. Le délai moyen de la prise en charge était de 7 jours.

**Figure 1:**
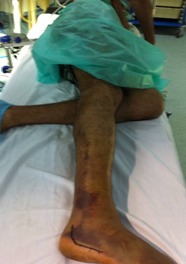
Installation du malade et voie d'abord

**Figure 2:**
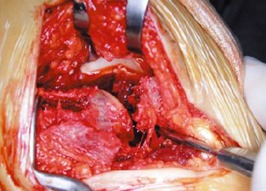
Vue per opératoire de la fracture

**Figure 3:**
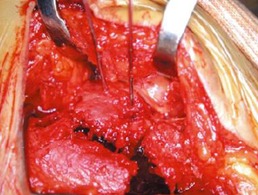
Rréduction provisoire de la fracture par broches

**Figure 4:**
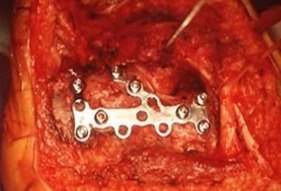
Vue per opératoire de la plaque anatomique

**Figure 5:**
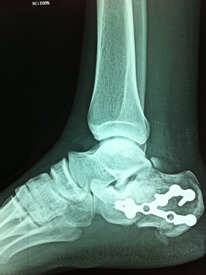
Contrôle post opératoire immédiat

La voie d abord utilisée était la voie externe sous et retro malléolaire en L (Figure 1). La dissection est menée jusqu'au périoste. Après L'analyse de la fracture et des surfaces articulaires sous talienne et Calcanéo-cuboïdienne (Figure 2), la réduction est obtenue par abaissement de la grosse tubérosité par un crochet et relèvement du fragment antéro-interne. Une fixation provisoire est entamée à l'aide de broches (Figure 3) puis s'en suit l'ostéosynthèse définitive par une plaque anatomique, mise sur la face externe du calcanéum (Figure 4, Figure 5). Deux de nos patients ont bénéficié d'une greffe cortico-spongieuse. Les malades ont été Immobilisés par une attelle plâtrée en botte pendant 45 jours. L'appui partiel a été autorisé à la 8ème semaine et n’était définitif qu'après 3 mois. En terme de complications, nous déplorons deux cas d'algodystrophie ayant évolué favorablement après rééducation, et deux cas de sepsis tardifs ayant nécessité une ablation du matériel d'ostéosynthèse. Un patient a présenté une arthrose sous talienne pour laquelle une arthrodèse a été proposée. Cette dernière a été refusée par le patient. Nos résultats fonctionnels ont été évalués selon le score de Kitaoka dont la moyenne était de 78 avec 68% de bon à très bon résultats: 7 patients (58%) présentaient une douleur occasionnelle contre 5 rapportant une indolence durable; la marche sur terrain irrégulier était normale chez 5 patients; la mobilité de la sous talienne était normale chez 7cas.

Sur le plan radiologique, l'angle de Bohler postopératoire moyen était de 27,25 (Tableau 1).

**Tableau 1:** Comparaison entre l'angle de Bohler en pré et en post opératoire immédiat

Angle de Bohler Pré-opératoire	Angle de Bohler Post-opératoire
-2°	28°
0°	30°
-10°	25°
-15°	12°
0°	30°
-5°	25°
0°	30°
-15°	2°
0°	28°
0°	30°
-15°	22°
5°	35°

## Discussion

Selon Eastwood, les fractures du calcanéum représentent 60% des fractures du tarse et l’étiologie principale étant la chute sur le talon [1]. Le symposium de la SOFCOT (société française d'orthopédie et de traumatologie) de 1988 préconisait une ostéosynthèse pour toutes les fractures articulaires déplacées [2]. Selon Babin l'ostéosynthèse par plaque, représente la meilleure technique opératoire [2]. Actuellement, la plupart des auteurs adoptent la même installation pour la même voie d'abord externe. Celle-ci, étant la plus recommandée, permet de bien contrôler les surfaces articulaires du calcanéum au dépend d'un risque de nécrose qui est de l'ordre de 21% selon Stephenson [3]. Ce risque pourrait être évité par une chirurgie différée entre le 7^ème^ et le 10^ème^ jour [4–6], règle qui était respectée chez nos malades et qui nous a épargné cette complication. Nous avons comparé nos résultats avec ceux de la littérature pour constater la supériorité de la plaque vissée surtout en montage triangulaire et anatomique, dans la restauration de l'angle de Bohler par rapport au vissage et à l'embrochage [7–9]. Cette restauration est durable lorsqu’ on respecte les zones d'ancrage des vis spongieuses définies par Babin et Saragaglia [10–12]. Dans ce sens, Stindel a retrouvé sur une série de 31 Cas traités par vissage et embrochage, un angle de Bohler postopératoire immédiat de 17 degré [6], alors que la correction était nettement meilleure dans les séries de Nouissri [13] et de Khourbi [14] utilisant la plaque vissée, traitant respectivement 42 et de 35 patients. Les valeurs oscillaient entre 16 et 28,5 degrés.

L'arthrose sous talienne constitue la complication la plus redoutable au long court. Elle est estimée à 5,6% dans la série de Zwipp comportant 194 patients [15]. Ce risque semble diminué dans les suites d'une ostéosynthèse par plaque [15]. Dans notre série, cette complication n'a pas pu être évaluée du fait de notre recul limité (18 mois). L'utilisation de greffon est laissée à l'appréciation de chaque auteur. Chez nos malades, cette option était jugée nécessaire dans deux cas, face à des défects osseux importants.

Nos résultats fonctionnels étaient comparables aux données de la littérature avec 68% de bon à très bon résultats soit un taux supérieur à celui des techniques utilisant les broche et les vis.

## Conclusion

Le traitement chirurgical des fractures articulaires du calcanéum permet de rétablir l'architecture triangulaire anatomique des travées osseuses principales. L'utilisation des plaques vissées adaptées apporte le meilleur résultat.
